# Establishment of Acquired Cisplatin Resistance in Ovarian Cancer Cell Lines Characterized by Enriched Metastatic Properties with Increased Twist Expression

**DOI:** 10.3390/ijms21207613

**Published:** 2020-10-15

**Authors:** Entaz Bahar, Ji-Ye Kim, Hyun-Soo Kim, Hyonok Yoon

**Affiliations:** 1 College of Pharmacy, Research Institute of Pharmaceutical Sciences, Gyeongsang National University, Jinju 52828, Korea; entazbahar@gnu.ac.kr; 2 Department of Pathology, Ilsan Paik Hospital, Inje University, Goyang 10380, Korea; alucion@gmail.com; 3 Department of Pathology, Yonsei University College of Medicine, Seoul 03722, Korea; 4 Department of Pathology and Translational Genomics, Samsung Medical Center, Sungkyunkwan University School of Medicine, Seoul 06351, Korea

**Keywords:** ovarian cancer, cisplatin resistance, Twist, metastasis, epithelial–mesenchymal transition

## Abstract

Ovarian cancer (OC) is the most lethal of the gynecologic cancers, and platinum-based treatment is a part of the standard first-line chemotherapy regimen. However, rapid development of acquired cisplatin resistance remains the main cause of treatment failure, and the underlying mechanism of resistance in OC treatment remains poorly understood. Faced with this problem, our aim in this study was to generate cisplatin-resistant (CisR) OC cell models in vitro and investigate the role of epithelial–mesenchymal transition (EMT) transcription factor Twist on acquired cisplatin resistance in OC cell models. To achieve this aim, OC cell lines OV-90 and SKOV-3 were exposed to cisplatin using pulse dosing and stepwise dose escalation methods for a duration of eight months, and a total of four CisR sublines were generated, two for each cell line. The acquired cisplatin resistance was confirmed by determination of 50% inhibitory concentration (IC_50_) and clonogenic survival assay. Furthermore, the CisR cells were studied to assess their respective characteristics of metastasis, EMT phenotype, DNA repair and endoplasmic reticulum stress-mediated cell death. We found the IC_50_ of CisR cells to cisplatin was 3–5 times higher than parental cells. The expression of Twist and metastatic ability of CisR cells were significantly greater than those of sensitive cells. The CisR cells displayed an EMT phenotype with decreased epithelial cell marker E-cadherin and increased mesenchymal proteins N-cadherin and vimentin. We observed that CisR cells showed significantly higher expression of DNA repair proteins, X-ray repair cross-complementing protein 1 (XRCC1) and poly (ADP-ribose) polymerases 1 (PARP1), with significantly reduced endoplasmic reticulum (ER) stress-mediated cell death. Moreover, Twist knockdown reduced metastatic ability of CisR cells by suppressing EMT, DNA repair and inducing ER stress-induced cell death. In conclusion, we highlighted the utilization of an acquired cisplatin resistance model to identify the potential role of Twist as a therapeutic target to reverse acquired cisplatin resistance in OC.

## 1. Introduction

The most lethal gynecological malignancy, ovarian cancer (OC) is the third most prevalent after cervical and uterine cancers and is the fifth leading cause of cancer-associated death in women worldwide [1,2]. Platinum–based chemotherapy has been the standard treatment for advanced OC for more than two decades [3,4,5,6]. Although significant treatment improvements have been achieved through platinum-based chemotherapy, for which the initial response rate is more than 80%, the 5-year survival rate for more than 75% of patients with advanced OC is only 15–25% [3,7]. One of the major factors contributing to this poor survival is the onset of cisplatin resistance. As such, there is an urgent need to understand the mechanisms of cisplatin resistance in OC.

Cisplatin resistance may be intrinsic due to inherited genetics or may be acquired following exposure to cisplatin [8,9,10]. The process of acquiring cisplatin resistance is not fully understood, but it is believed that tumors resistances to cisplatin are also resistance to the other platinum drugs [11,12]. It has been demonstrated that acquired cisplatin resistance is associated with overexpression of ATP-binding cassette (ABC) transporters, reduced drug accumulation, insufficient DNA binding, increased DNA repair, and altered expression and activation of genes involved in cell death pathways [9,13,14,15,16,17,18]. Some studies have suggested a strong correlation between epithelial–mesenchymal transition (EMT) and drug resistance [19,20]. Recently, it has been reported that cisplatin-resistant oral squamous cell carcinoma cells upregulate the activity of multidrug resistance protein 1 (MDR1) or breast cancer resistance protein (BCRP) associated with EMT [21].

Several studies have revealed that multiple complex mechanisms are involved in acquired cisplatin resistance [11,12,17]. However, despite the notable progress made toward understanding drug-resistant cancer at the molecular and cellular levels, our knowledge of the biological pathways involved in acquired cisplatin resistance remains limited due to a lack of experimental tools. The establishment of cisplatin-resistant (CisR) human cancer cell lines may be a convenient and effective model system to investigate the molecular mechanisms of cancer drug resistance [22]. The two most common methods to establish resistance cell lines described in literature are high-concentration pulsatile exposure (short duration/high dose) and stepwise dose-escalation continuous (long duration/low dose) [23,24]. Both methods differ in their resistance mechanism and each has its own advantages. The stepwise dose increment method is more commonly used due to higher success rate because long-term treatment generates cell lines with greater stability [25,26]. However, pulsatile treatment generates resistant cell lines by applying selective pressure for cell clones with intrinsic resistant mutations. Pulsatile treatment is also considered to most closely simulate the clinical therapy protocol administered to a patient. Human OC-derived cell lines, selected by exposure to cisplatin, have been valuable tools to identify the principal factors fundamental to in vitro acquired cisplatin resistance. 

The helix–loop–helix transcriptional factor Twist, a master regulator of EMT, has been associated with highly invasive and metastasis carcinoma and correlated to the development of tumor recurrence and chemoresistance [27,28,29,30,31,32,33,34]. In several cancer types, Twist has been linked to resistance to the platinum-based chemotherapy, including cisplatin as well as paclitaxel and doxorubicin [35,36,37,38,39,40]. Moreover, a few studies have been linked to the development of platinum resistance and Twist expression, yet little is known regarding the role of Twist on platinum resistance in OC. Therefore, the goal of this study was to establish CisR OC cell lines and investigate the role of Twist in OC cell metastasis and cisplatin resistance. 

## 2. Results

### 2.1. Generation and Characterization of Cisplatin Resistance in Ovarian Cancer Cell Lines

#### 2.1.1. Generation of Cisplatin Resistance in (CisR) Ovarian Cancer (OC) Cell Lines

**Figure 1:**
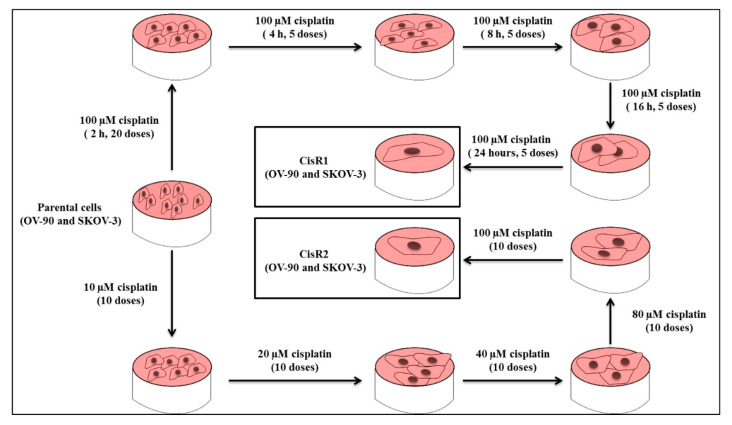
Generation of acquired cisplatin resistance OV-90 and SKOV-3 ovarian cancer (OC) cell lines. Two sublines were generated from each cell line, using pulse and intermittent treatment method, namely CisR1 (SKOV-3/CisR1 and OV-90/CisR1) and CisR2 (SKOV-3/CisR2 and OV-90/CisR2).

To generate CisR OC cell lines, we selected two OC cell lines including the high-grade serous epithelial OC model cell line, OV-90, and the mixed epithelial OC cell line, SKOV-3 [41,42]. We used a constant higher dose (100 µM) of cisplatin for pulse treatment and started from a lower dose (10, 20, 40, 80 to 100 µM) of cisplatin for intermittent incremental treatment methods to generate CisR OC cells. A total of four sublines were generated, two from each cell line, including OV-90/CisR1, OV-90/CisR2, SKOV-3/CisR1 and SKOV-3/CisR2 (Figure 1).

**Figure 2:**
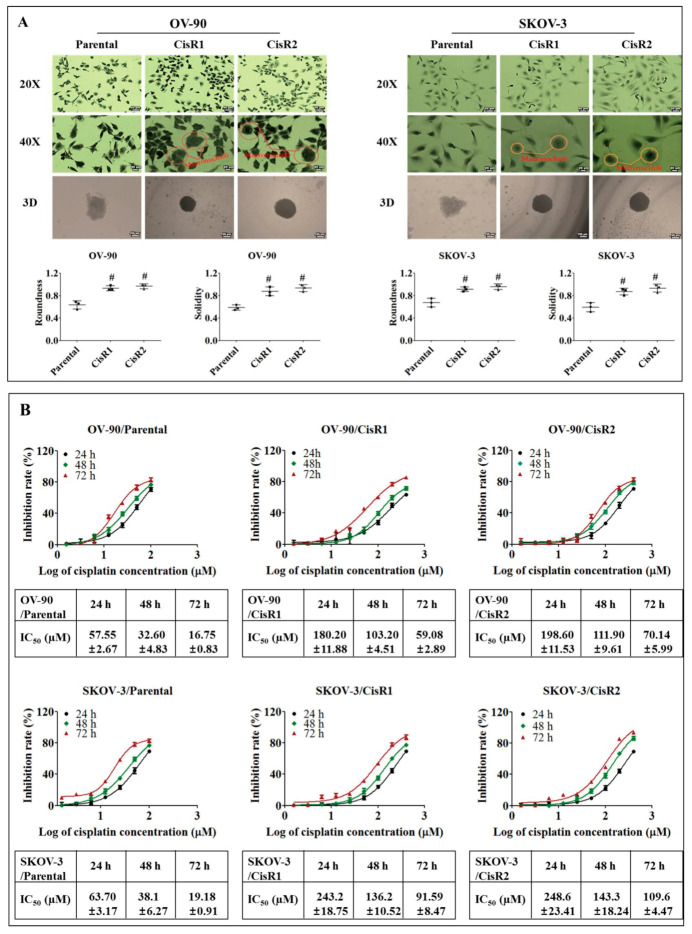
Characterization of acquired CisR OC cells by measurement of 50% inhibitory concentration (IC_50_). (**A**) Morphological evaluation of CisR and parental cell lines by 2-dimensional (2D) (Magnification, upper panel 20×, scale bar 50 µm; middle panel 40×, scale bar 20 µm) and 3-dimensional (3D) (Magnification, lower panel 10×, scale bar 100 µm) cell culture. (**B**) The IC_50_ values were evaluated for parental and CisR OC cells by measuring 50% inhibition of cisplatin at 24, 48 and 72 h. Values were represented as mean ± SD (*n* = 3). ^#^ *p* < 0.05, compared with the parental group.

On morphological evaluation, the parental cells displayed a polygonal shape with more regular shapes and sizes and were attached to the culture dish in discrete clusters, where CisR cells had variation in cell sizes, occasional enlarged multinucleated “giant” cells, prominent macronucleoli, and increased number of cellular processes (dendrites). The CisR cells also demonstrated stronger adhesion to the culture dish than parental cells (Figure 2A).

Furthermore, the parental and CisR OC cells were analyzed for spheroid formation capacity in Poly-HEMA coated 12-well plates by utilizing hanging drop method. CisR cells exhibited more cancer stem cell (CSC)-like characteristics than their parental OC cells. The spheroids in CisR cells were more round, solid and tightly compact compared to their parental cells (Figure 2A).

Inhibitory concentration (IC_50_) values were evaluated for parental and CisR cells by measuring the percentage of inhibition of cisplatin at 24, 48 and 72 h. It was observed that a significant increase in the dose of cisplatin was required to inhibit 50% of cell growth in both CisR cells compared to their corresponding parental cells (Figure 2B). The IC_50_ values of cisplatin in the OV-90/parental cell line were 57.55 ± 2.67, 32.60 ± 4.83, 16.75 ± 0.83 µM at 24, 48 and 72 h, respectively. However, the IC_50_ values in OV-90/CisR1 and OV-90/CisR2 were 180.2 ± 11.88, 103.2± 4.51, 59.08 ± 2.89 and 198.6 ± 11.53, 111.3 ± 9.61, 70.14 ± 5.99 µM, respectively, at 24, 48 and 72 h. Similarly, in SKOV-3 cell line, a significant increase in the IC_50_ values of CisR cells was observed. The IC_50_ values of cisplatin in SKOV3/parental cell were 63.70 ± 3.17, 38.13 ± 6.27, 19.18 ± 0.91 µM at 24, 48 and 72h, respectively. The IC_50_ values in SKOV-3/CisR1 and SKOV-3/CisR2 were 243.2 ± 18.75, 136.2 ± 10.52, 91.59 ± 8.468, and 248.5 ± 23.41, 143.3 ± 18.24, 109.6 ± 1.47 µM, respectively, at 24, 48 and 72 h. From the dose–response curve, a significant increase in IC_50_ values of cisplatin was observed in CisR cells, OV-90/CisR1 cells (59.08 ± 2.89 µM vs. 16.75 ± 0.83 µM), OV-90/CisR2 (70.14 ± 5.99 µM vs. 16.75 ± 0.83 µM) at 72 h, which showed a 3.53-fold (OV-90/CisR1) and 4.19-fold (OV-90/CisR2) increase in the concentration of cisplatin required to obtain a 50% inhibition in cell growth (Figure S1A). In SKOV-3 cells, the IC_50_ values of CisR cells, SKOV-3/CisR1 and SKOV-3/CisR2, were determined as 91.59 ± 8.47 and 109.6 ± 4.47 µM, respectively, compared to 19.18 ± 0.91 µM for 72 h in the original parent cell line, which was a 4.77-fold (SKOV-3/CisR1) and 5.71-fold (SKOV-3/CisR2) increase in the concentration of cisplatin required to obtain a 50% inhibition in cell growth (Figure S1B).

#### 2.1.2. The CisR OC Cells Display Higher Twist Expression with Increased Metastasis Abilities than Their Parental OC Cells

**Figure 3:**
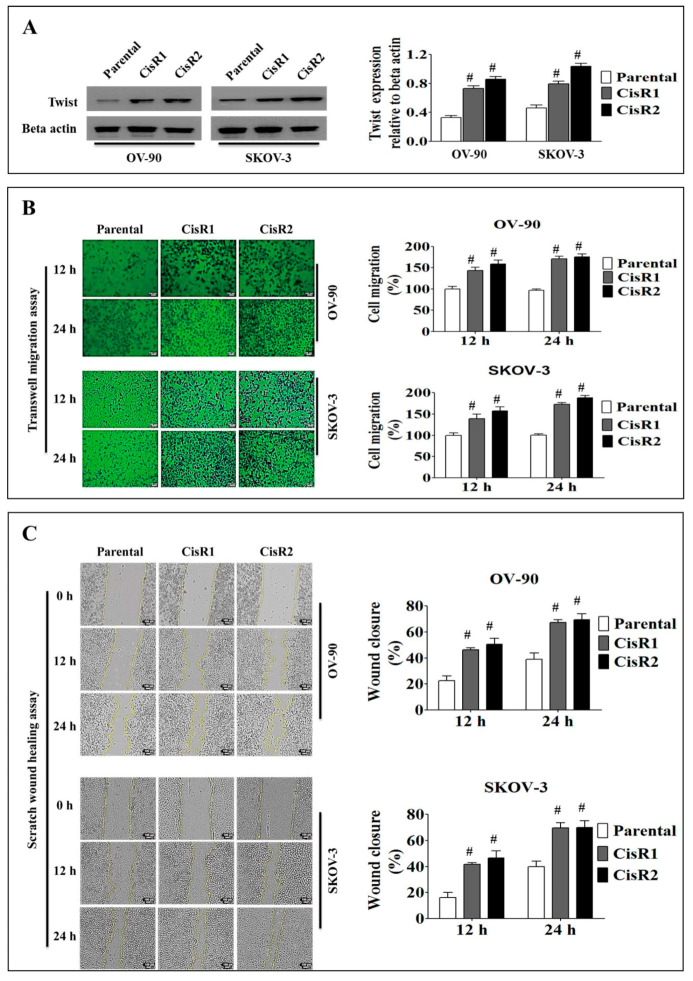
Metastasis behavior of cisplatin-resistant and parental OC cells. (**A**) Twist expression in CisR and parental OC cells. (**B**) Migration ability of cisplatin-resistant and parental OC cells determined by transwell migration assay (Magnification, 10×, scale bar 100 µm). (**C**) Wound healing capability of CisR and parental OC cells (Magnification, 10×, scale bar 100 µm). Values were represented as mean ± SD (*n* = 3). ^#^ *p* < 0.05, compared with the parental group.

We observed significant increased Twist expression level in OV-90/CisR1, OV-90/CisR2, SKOV-3/CisR1 and SKOV-3/CisR2 OC cells compared to their parental cells (Figure 3A). The transwell migration assays revealed that CisR cells had greater migration abilities compared to parental cells at 12 and 24 h (Figure 3B). Similarly, the CisR cells exhibited greater wound healing ability relative to parental cell in wound healing assay at 12 and 24 h (Figure 3C). 

In transwell migration assay, CisR cells showed significantly higher migration rates compared to parental cells (Figure 3B). The migration speed was higher in CisR sublines compared to parental cells at 12 and 24 h (Figure S2A). In OV-90 cells, the CisR sublines showed higher average migration speed, in OV-90/CisR1 (9.52 ± 0.45%/h vs. 6.18 ± 0.32%/h), OV-90/CisR2 (10.25 ± 0.55%/h vs. 6.18 ± 0.32%/h), SKOV-3/CisR1 (9.29 ± 0.40%/h vs. 6.25 ± 0.39%/h) and SKOV-3/CisR2 (10.78 ± 0.59%/h vs. 6.25 ± 0.39%/h) (Figure S2B).

In wound healing assay, CisR cells showed significantly greater wound healing ability compared to parental cells (Figure 3C). The wound closure rate was higher in CisR than in parental cells at 12 and 24 h. The CisR cells showed a significantly higher average wound closure rate, in OV-90/CisR (37.76 ± 1.39% vs. 20.51 ± 3.09% of parental), OV-90/CisR2 (40.97 ± 3.8% vs. 20.51 ± 3.09% of parental), SKOV-3/CisR1 (37.081 ± 1.70% vs. 18.56 ± 2.88% of parental) and SKOV-3/CisR2 (38.94 ± 3.8% vs. 18.56 ± 2.88% of parental) (Figure S3A). The relative wound width (area) was smaller in CisR cells compared to the parental cell (Figure S3B). The wound healing speed was higher in CisR sublines compared to parental cells at 12 and 24 h and showed an upward curve up to 12 h (Figure S3C). The CisR sublines showed higher average wound healing speed, in OV-90/CisR1 (36,852.04 ± 1358.73 µm^2^/h vs. 14,339.6291 ± 2901.75 µm^2^/h), OV-90/CisR2 (41,691.87 ± 3552.77 µm^2^/h vs. 14,339.6291 ± 2901.75 µm^2^/h), SKOV-3/CisR1(38,823.93 ± 1627.29 µm^2^/h vs. 18,385.69 ± 3298.35 µm^2^/h) and SKOV-3/CisR2 (42,954.47 ± 51,119.76 µm^2^/h vs. 18,385.69 ± 3298.35 µm^2^/h) (Figure S3D).

Taken together, these initial data revealed that a cisplatin-resistant phenotype in four OC sublines induced metastatic signals of increasing cell migration ability following chronic in vitro exposure to cisplatin.

#### 2.1.3. The CisR OC Cells Exhibit Higher Metastasis Ability with an EMT Phenotype

**Figure 4:**
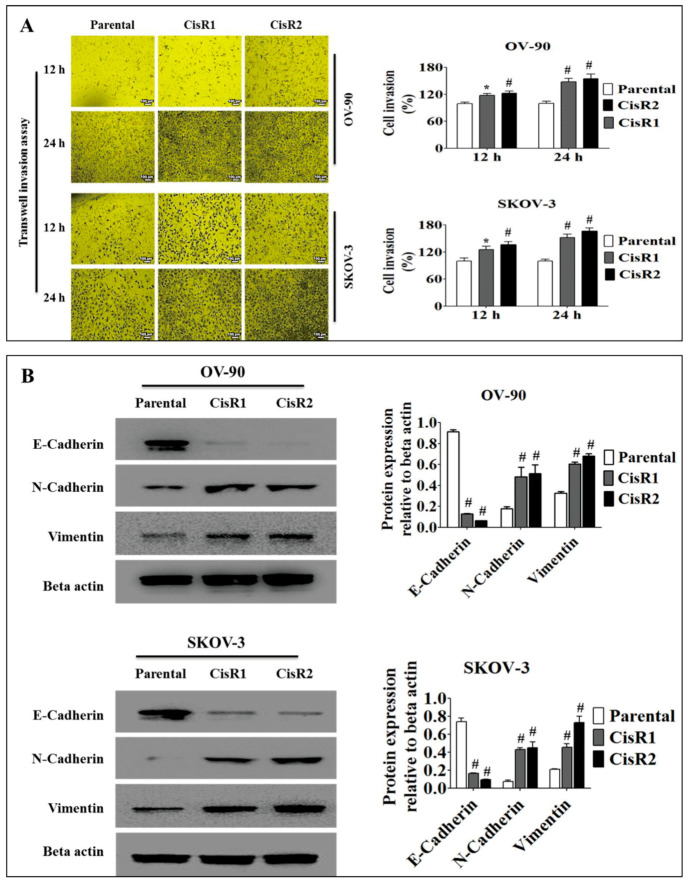
The involvement of epithelial–mesenchymal transition (EMT) in acquired CisR and parental OC cells. (**A**) Transwell invasion assay of parental versus CisR OV-90 and SKOV-3 OC cells (Magnification, 10×, scale bar 100 µm). (**B**) Western blot analysis of EMT markers, E-cadherin, N-cadherin and Vimentin. Values were represented as mean ± SD (*n* = 3). * *p* < 0.05, ^#^ *p* < 0.01, compared with the parental group.

To confirm the occurrence of EMT in CisR OC cells, we evaluated cell invasion and adhesion assay to confirm cell invasive and cell-extracellular matrix (ECM) adhesion capability. To accomplish this, the parental and CisR OC cells were each treated with 50 µm of cisplatin for 24 h. In invasion assay, the CisR cells showed increased levels of invasion compared to parental cells (Figure 4A). The invasion rate was significantly higher in CisR sublines compared to parental cells at 12 and 24 h, where up to 12 h it showed an upward curve (Figure S4A). The CisR sublines showed higher average invasion speed, in OV-90/CisR1 (7.91 ± 0.20%/h vs. 6.26 ± 0.39%/h), OV-90/CisR2 (8.43 ± 0.27%/h vs. 6.26 ± 0.39%/h), SKOV-3/CisR1 (8.54 ± 0.38%/h vs. 6.25 ± 0.37%/h) and SKOV-3/CisR2 (9.14 ± 0.44%/h vs. 6.25 ± 0.37%/h) (Figure S4B). 

Our results demonstrated that the extracellular matrix (ECM)-like fibronectin was associated with a decreased sensitivity to cisplatin-based drug treatment, as CisR cells displayed higher cell-ECM adhesion compared to the parental OC cells (Figure S5). In OV-90 cells, the cell-ECM adhesion rate was higher in CisR sublines, OV-90/CisR1 (133.25 ± 3.7% vs.% of parental) and OV-90/CisR2 (141.4 ± 3.5% vs.% of parental). In SKOV-3 cells, the cell-ECM adhesion rate was also higher in CisR sublines, SKOV-3/CisR1 (148.2 ± 3.5% vs.% of parental) and SKOV-3/CisR2 (154.6 ± 3.4% vs.% of parental). For further confirmation, we examined the most common epithelial (E-cadherin) and mesenchymal (N-cadherin and vimentin) markers. Our Western blot data demonstrated that CisR OC cells displayed EMT phenotype (Figure 4B). The expression level of the epithelial marker E-cadherin was lower in CisR cells compared to parental cell. However, the CisR cells showed significantly higher expression of mesenchymal marker proteins, N-cadherin and vimentin. Taken together, these results demonstrated that chronic exposure to cisplatin induced metastatic signals leading to EMT phenotypes.

#### 2.1.4. Acquisition of Cisplatin Resistance in OC Results in Higher Cell Colonization and Poor Apoptotic Signatures Associated with Activation of the DNA Excision Repair Pathway and Suppression of ER Stress Mediated Apoptosis

**Figure 5:**
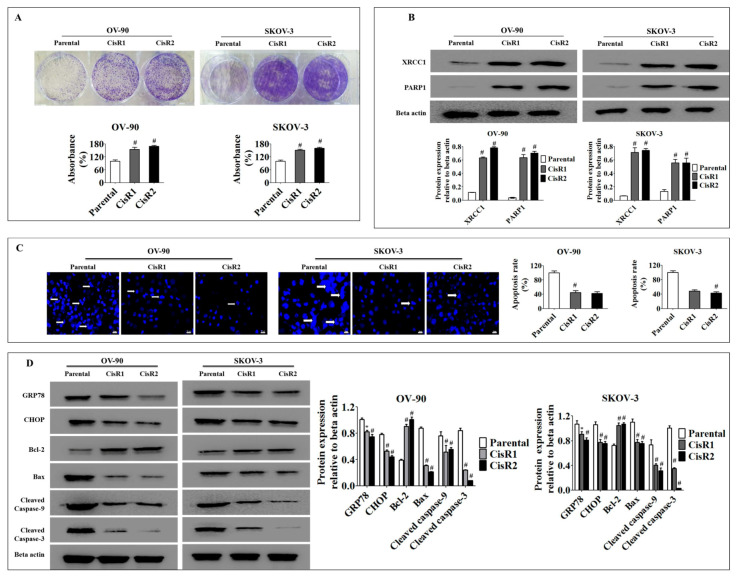
The CisR OC activated DNA repair pathways and suppressed endoplasmic reticulum (ER)-stress mediated cell death. (**A**) The clonogenic growth rate analysis reveals a significantly faster clonogenic growth of CisR cells compared to parental cells. (**B**) Western blot analysis of DNA repair proteins, XRCC1 and PARP1, demonstrating significantly higher expression of DNA repair proteins in CisR cells compared to parental cells. (**C**) Apoptosis rate evaluated by Hoechst33342 staining shows significantly lower apoptosis in CisR cells compared to parental cells (Magnification, 40×, scale bar 20 µm) (**D**) the Western blot analysis for GRP78, CHOP, Bcl-2, Bax, cleaved caspase-9 and cleaved caspase-3 shows significantly decreased levels of pro-apoptotic proteins and significantly increased levels of anti-apoptotic protein in CisR compared to parental cells. Values were represented as mean ± SD. * *p* < 0.05, ^#^ *p* < 0.01, compared with the parental group.

XRCC1 and PARP1 deficient cancer cells have been demonstrated to show greater sensitivity to cisplatin [43]. Thus, we investigated whether acquired cisplatin resistance was associated with a significant change in DNA repair proteins. We examined the clonogenic growth rate of both cisplatin resistance and parental OC cell lines to confirm survival capability. The CisR cells had increased clonogenic activity as early as 24 h of cisplatin incubation followed by the ninth day of recovery period compared to parental OC cells (Figure 5A). Furthermore, our Western blot analysis revealed that CisR cells significantly altered DNA repair protein (XRCC1 and PARP1) expression compared to parental OC cells (Figure 5B), which could enhance survival by repairing the cisplatin-damaged DNA and rescuing the cell from apoptosis.

Since many chemotherapeutic drugs, including cisplatin, cause cell death by inducing endoplasmic reticulum (ER)-stress-mediated apoptosis, an altered ER stress-dependent apoptotic response diminishes the efficacy of these drugs in resistant cells [18,44,45,46,47]. Studies on colon cancer, breast cancer and osteosarcoma have demonstrated that acquired chemotherapy resistant cancer cells have resistance to ER stress-triggered cell death [18,48,49]. Thus, we evaluated whether ER stress-mediated apoptosis is involved in acquired cisplatin resistance in OC cells. To accomplish this, both the parental and the CisR OC cells were treated with 50 µM of cisplatin for 24 h. Our results revealed that CisR cells had a poor apoptotic signature profile with a reduced level of apoptotic proteins (Bax, cleaved caspase-9 and cleaved caspase-3) and an increased level of anti-apoptotic protein (Bcl-2) compared to parental OC cells (Figure 5C,D) [50]. Subsequently, cisplatin significantly increased the expression of molecular markers of ER stress, such as GRP78, CHOP in parental OC cells compared to CisR OC cells [51,52]. Taken together, these findings demonstrate that cisplatin-induced ER stress-mediated apoptosis was significantly diminished in the CisR cells compared to the parental OC cells, which may point to an important underlying mechanism of CisR OC to avoid cellular death by cisplatin.

### 2.2. Twist Knockdown Can Affect the Metastasis Potential of Acquired CisR OC Cells

**Figure 6:**
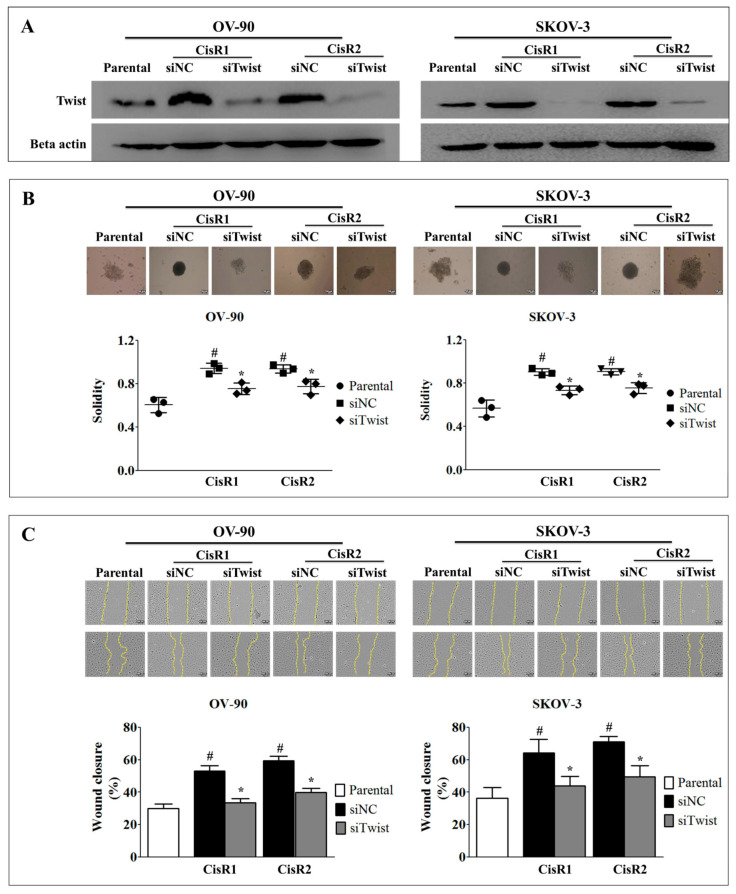
Twist knockdown result in reduction of metastasis properties in CisR OC cells. (**A**) Confirmation of Twist knockdown by western blot analysis. (**B**) 3-dimensional spheroid formation analysis of Twist knockdown CisR OC cells (Magnification, 10×, scale bar 100 µm). (**C**) Wound healing potential of Twist knockdown CisR OC cells (Magnification, 10×, scale bar 100 µm). Parental: Non-transfected parental cells; siNC: Cisplatin resistance cells transfected with non-targeting negative control siRNA; siTwist: Cisplatin resistance cells transfected with Twist siRNA. Values were represented as mean ± SD. ^#^ *p* < 0.05, compared with the parental group and * *p* < 0.05, compared with siNC group.

To investigate the role of Twist in cisplatin resistance, we generated the Twist knockdown (siTwist) OV-90/CisR1, OV-90/CisR2, SKOV-3/CisR1 and SKOV-3/CisR2 OC cells and siRNA negative control (siNC) cells (Figure 6A). In order to justify re-sanitization capacity of CisR cells by Twist knockdown, we included non-transfected parental cell in our experiment. The siTwist cells significantly changed 3D spheroid formation capacity than siNC. The siTwist showed reduced spheroid roundness and solidity than siNC (Figure 6B). The siTwist cells displayed the reduced wound healing capacity then siNC (Figure 6C).

### 2.3. Twist Knockdown Attenuated Cell Metastasis Properties via Suppression of Cell Invasion and EMT Phenotype in Acquired CisR OC Cells

**Figure 7:**
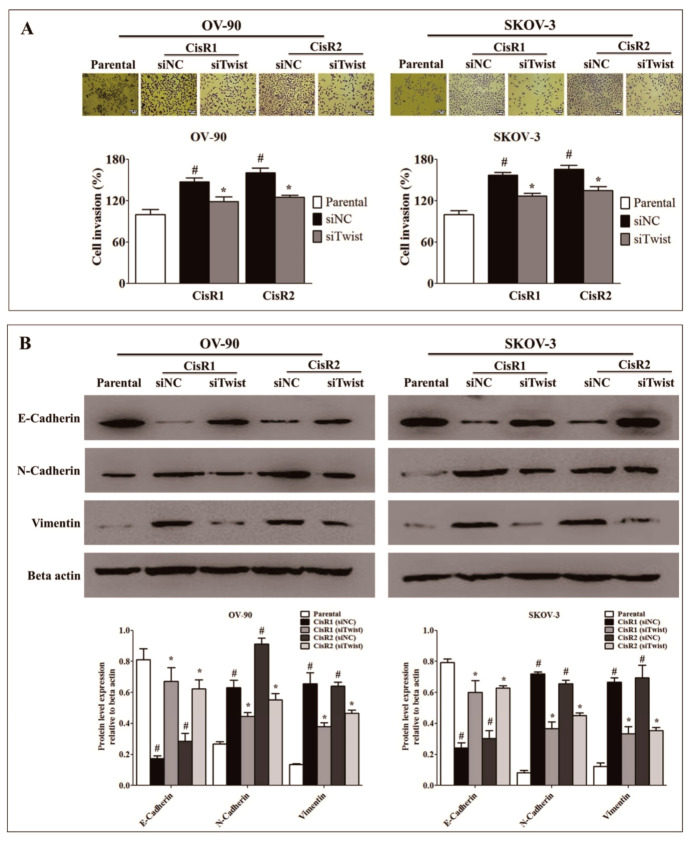
Twist knockdown reverses invasion and EMT phenotype. (**A**) Invasion ability of Twist knockdown CisR OC cells (Magnification, 10×, scale bar 100 µm). (**B**) The effect of Twist knockdown on expression of EMT-related proteins, E-cadherin, N-cadherin and vimentin. Parental: non-transfected parental cells; siNC: cisplatin resistance cells transfected with non-targeting negative control siRNA; siTwist: cisplatin resistance cells transfected with Twist siRNA. Values were represented as mean ± SD. ^#^ *p* < 0.05, compared with the parental group and * *p* < 0.05, compared with siNC group.

To investigate the molecular mechanism by which Twist mediated cisplatin resistance, we investigated cell metastasis capacity by invasion and the expression level of EMT-related proteins by Western blot. The siTwist cells showed lower cell invasion (Figure 7A) ability than siNC cells. The expression of epithelial cell marker protein, E-cadherin was significantly increased in siTwist cells than siNC cells, while N-cadherin and vimentin, mesenchymal cell marker significantly downregulated in siTwist than siNC (Figure 7B).

### 2.4. Twist Knockdown Reduces Cell Survival Potential via Downregulation of DNA Repair Pathway and Activation of ER-Stress-Mediated Cell Death in CisR OC Cells

**Figure 8:**
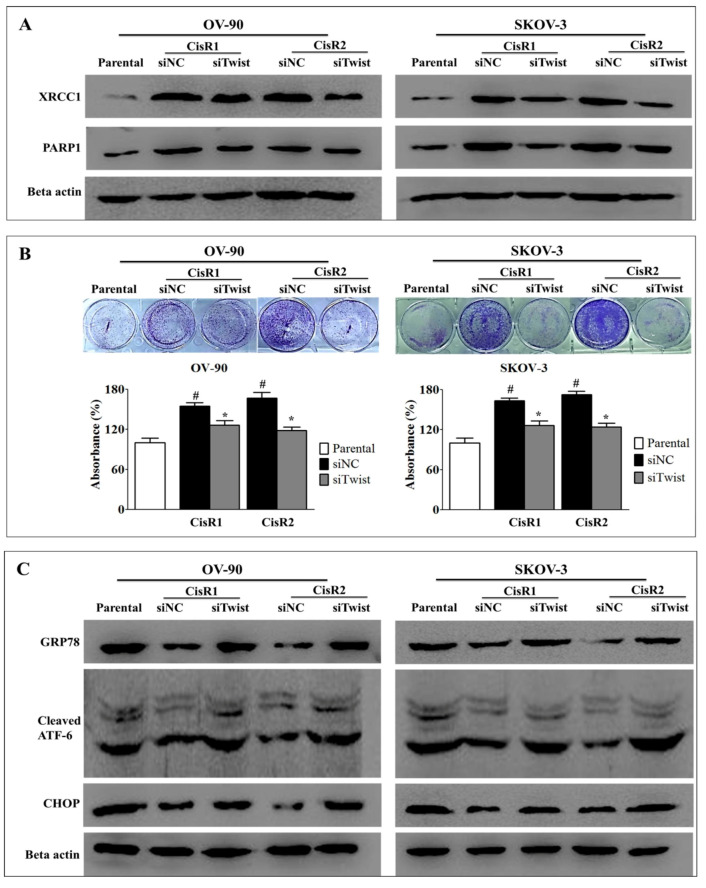
Twist knockdown reduces cell growth potential in CisR OC cells. (**A**) Western blot analysis for DNA repair proteins, PARP1 and XRCC1. (**B**) The cell survival analysis assayed by clonogenic assay. (**C**) Western blot analysis for expression of ER stress signaling proteins, GRP78, cleaved ATF-6 and CHOP. Parental: non-transfected parental cells; siNC: cisplatin resistance cells transfected with non-targeting negative control siRNA; siTwist: cisplatin resistance cells transfected with Twist siRNA. Values were represented as mean ± SD. ^#^ *p* < 0.05, compared with the parental group and * *p* < 0.05, compared with siNC group.

We observed that the expression of DNA repair proteins-PARP1 and XRCC1 (Figure 8A and Figure S7) and the cell survival capacity (Figure 8B) were lower in siTwist cells than siNC cells. Twist knockdown increased the ER stress response in CisR cells, as indicated by significant increase in the expression of GRP78, cleaved ATF-6 and CHOP (Figure 8C and Figure S7). 

**Figure 9:**
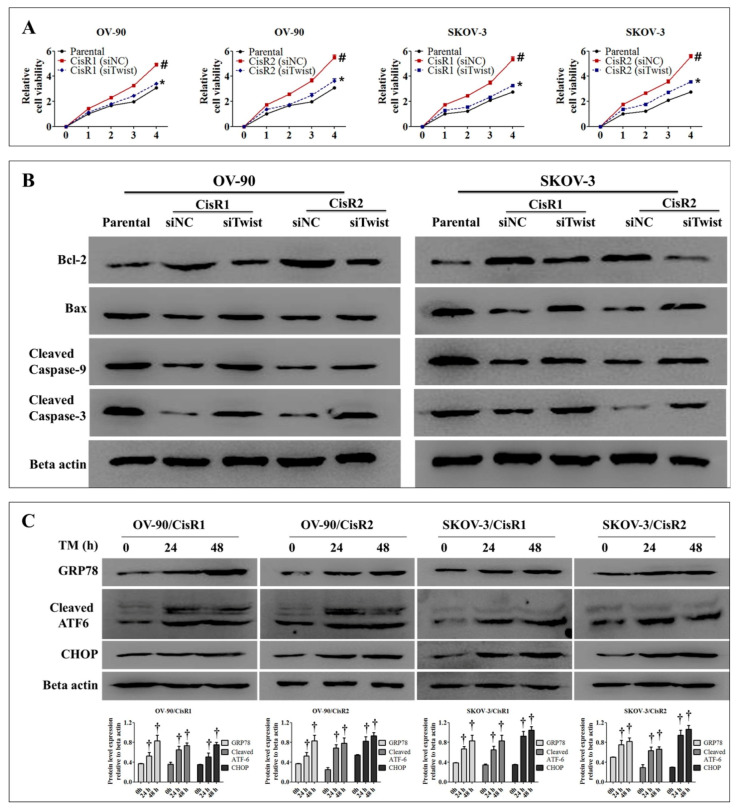
Twist knockdown induces cell death in CisR OC cells. (**A**) The relative cell viability. (**B**) Western blot analysis for anti-apoptotic protein, Bcl-2 and apoptotic proteins, Bax, cleaved caspase-9 and cleaved caspase-3. (**C**) Tunicamycin-induced ER stress response in CisR cells assayed by Western blot analysis of GRP78, cleaved ATF-6 and CHOP. Parental: non-transfected parental cells; siNC: cisplatin resistance cells transfected with non-targeting negative control siRNA; siTwist: cisplatin resistance cells transfected with Twist siRNA. Values were represented as mean ± SD. ^#^ *p* < 0.05, compared with the parental group, * *p* < 0.05, compared with siNC group and ^†^ *p* < 0.05, compared with 0 h of CisR group.

The result also showed that knockdown of Twist in CisR cells significantly reduced relative cell viability (Figure 9A) and induced the magnitude of cell death by downregulation of regulation anti-apoptotic protein Bcl-2 and upregulation of apoptotic proteins Bax, cleaved caspase-9 and cleaved caspase-3 (Figure 9B and Figure S8).

Under ER stress, cellular dysfunction and cell death often occurred. As expected, the CisR cells were treated with tunicamycin (5 µg/mL) for 48 h to induce ER stress. The upregulation of GRP78, cleaved ATF-6 and CHOP were observed at 24 and 48 h, as indicated elevation of ER stress (Figure 9C). 

### 2.5. ER Stress Inhibition Reversed the Twist Knockdown-Induced Cell Death

**Figure 10:**
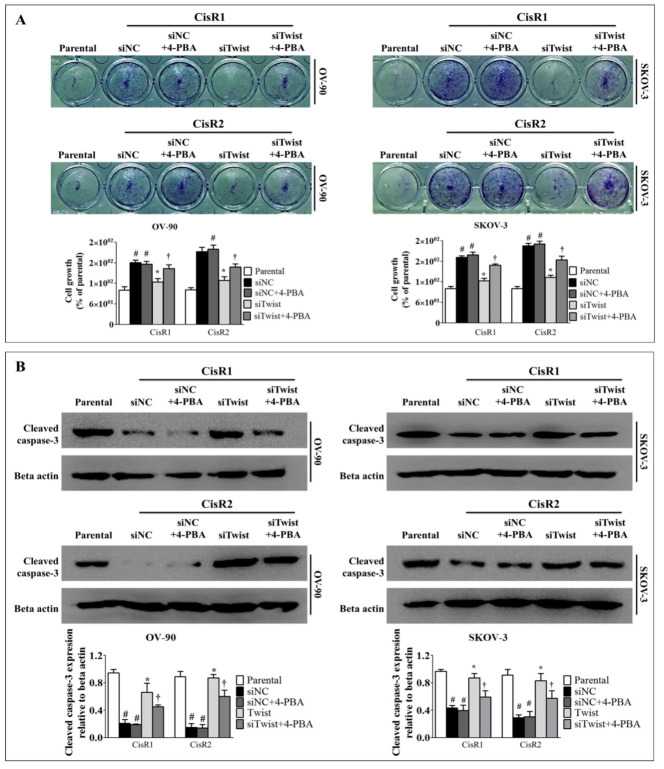
ER stress inhibition reversed the Twist knockdown-induced cell death. (**A**) The cell growth analysis. (**B**) Western blot analysis apoptotic protein, cleaved caspase-3. The CisR cells were treated with 2.5 mM 4-PBA for 5 days. Parental: non-transfected parental cells; siNC: cisplatin resistance cells transfected with non-targeting negative control siRNA; siTwist: cisplatin resistance cells transfected with Twist siRNA. Values were represented as mean ± SD. ^#^ *p* < 0.05, compared with the parental group, * *p* < 0.05, compared with siNC group and ^†^ *p* < 0.05, compared with siTwist group.

To investigate the implication of ER stress, we used 2.5 mM of 4-phenylbutyric acid (4-PBA) to diminish ER stress. The knockdown of Twist reduced cell growth and induced cell death (Figure 8B and Figure 9A,B). Interestingly, 4-PBA treatment reversed the Twist knockdown-induced cell growth (Figure 10A), and also rescued the CisR cells from Twist knockdown-induced cell death by attenuating apoptotic protein cleaved caspase-3 expression (Figure 10B). These results represented how Twist knockdown acted via ER stress to induce cell death in CisR OC cells.

## 3. Discussion

In this study, we established CisR OC models using high dose pulse treatment and a stepwise increasing dose of cisplatin to characterize the evolution of acquired resistance during cisplatin-based anti-cancer therapy. The pulse dosing is identical to that used in hospital treatment with a pulse therapy regimen, using a high dose of the chemotherapy drug followed by a rest period calculated to allow the patient to recover from adverse effects [53,54]. The treatment method using stepwise drug dose escalation can be clinically effective for an oral drug given daily or twice daily, as a relatively constant amount of the drug is present within the body [24,55]. When establishing resistant cell lines, the pulse dosing method has generally been considered as inferior compared to the intermittent incremental method due to relatively lesser stability and strength of drug resistance [24,56,57]. In our study, we established a genetically stable and clinically relevant CisR OC cell by modifying the conventional pulse dosing method with gradually increasing duration of drug incubation period with constant high dose [58]. 

In ovarian cancer research, in vitro cell line models become effective tools to understand the molecular mechanisms underlying acquired chemo-resistance development in ovarian cancer [59,60,61,62]. We derived and confirmed the acquired cisplatin resistance cell model through both pulse and stepwise dose increasing methods, which offer a useful tool for describing the molecular mechanisms of acquired cisplatin resistance. Morphologically, the CisR cells were different from the parental OC cell. CisR cell lines exhibited, multinucleated “giant” cells, greater variability in cell size, and prominent macronucleoli compared to the parental OC cell. These CisR cells were confirmed with cisplatin IC_50_ values at least 3–5 times greater than those of the corresponding parental cell.

Chemotherapy resistance with metastasis is a major obstacle to successful cancer treatment [63,64]. Epithelial OC patients receiving chemotherapy usually develop acquired drug resistance within one year, which leads to tumor recurrence and uncontrolled metastases [65]. Metastasis, one of the most important hallmarks of malignancy, is a complex process that involves migration and invasion of cancer cells [66,67,68]. Several studies have suggested that chemotherapy resistance is acquired by the metastatic growth of tumor cells that may closely parallel each other [69,70,71]. It has been demonstrated that metastasis-related genes play a vital role in cisplatin chemo-resistance [72]. Our results suggested that CisR in OC cells altered their characteristics and retained the majority of metastatic properties by increasing the cell proliferation, cancer stem cell (CSC)-like characteristics, migration and invasion abilities of cancer cells [73]. 

DNA nucleotide excision repair (NER) and base excision repair (BER) are the most common DNA repair mechanisms that arise to repair DNA damage caused by cisplatin [74,75,76,77,78]. Poly (ADP-ribose) polymerases 1 (PARP1) interacts with X-ray repair cross-complementing protein 1 (XRCC1) to trigger the BER DNA repair process [79,80,81,82]. NER and BER are the mechanisms in repairing the DNA crosslink induced by cisplatin [83,84]. Therefore, DNA repair proteins, XRCC1 and PARP1, have been associated with significantly aggressive clinical outcome of ovarian cancer patients and decreased cisplatin sensitivity in OC cells. XRCC1 is a scaffolding protein that interacts with BER factors, including Ligase III, DNA polymerase β and PARP1, to recruit them to the DNA breaks, thus vital to BER [85]. PARP1 is an important protein that is recruited to the site of DNA damage to trigger poly (ADP-ribosylation) of multiple substrates, which leads to the activation of DNA repair [81]. On such basis, PARP inhibitors have been clinically effective for their anti-cancer effects [86,87]. The results of our study are also consistent with previous studies because the significantly increased level of XRCC1 and PARP1 correlated with cisplatin resistance in both SKOV-3 and OV-90. Taken together, these results support our findings that XRCC1 and PARP1 are important to the development of cisplatin resistance in OC.

We observed that in CisR OC cells had significantly decreased apoptotic proteins of the ER stress pathway and mitochondrial pathway compared to parental OC cells. Anti-apoptotic protein Bcl2 in CisR OC cells were significantly increased compared to parental cells, in agreement with other previous in vitro studies involving CisR cell lines [88]. However, our observations of significantly decreased GRP78 protein level conflict with previous reports in other malignancies showing that GRP78 exerts pro-survival and chemo-resistant effects. GRP78 is an important chaperone protein of the ER which has been implicated in cancer resistance against chemotherapy involving apoptotic pathways. This has been demonstrated through increased sensitivity against therapeutic drugs by knockdown of GRP78 in glioblastoma. In addition, GRP78 has been associated with poor survival in breast, liver, prostate, colon and gastric cancers with the exception of lung cancer [89,90,91,92,93]. These conflicting data regarding the role of GRP78 may be related to the differences in organs and cell types, as well as the inadequacy of using a single model to explain the complex process of cisplatin resistance. 

EMT, a hallmark of aggressive and highly invasive cancers, contributes to CisR in OC cells by suppressing the epithelial marker (E-cadherin) and enhancing the expression of mesenchymal marker proteins (N-cadherin and vimentin) [94]. The EMT of cancer cells has been considered to be an important mechanism for cancer metastasis and chemotherapy resistance [65,95]. EMT-associated proteins are highly expressed in chemotherapy resistance and have been associated with enhanced migration and metastasis of tumor cells [96,97,98]. Many studies have demonstrated that chemotherapy resistance and metastasis are controlled by reversible phenotypic transitions between epithelial and mesenchymal phenotypes (EMT and MET), which is referred to as epithelial plasticity [99,100,101,102,103]. The tumor cells can also reach a state of partial or intermediate EMT during transitioning between EMT and MET, called hybrid epithelial/mesenchymal phenotype [95,104,105].

One of the important events contributing to EMT is the activation of EMT- transcription factors (TFs), such as Twist that act as repressors for epithelial genes and as activators for mesenchymal genes [106,107,108,109]. We demonstrated significant increased levels of Twist, N-cadherin and vimentin and significantly decreased levels of E-cadherin in CisR cells compared to parental cells. This was in agreement with studies that noted that EMT-TFs that regulated EMT, such as Twist, have been demonstrated to mediate the development of cancer cell resistance to platinum-based anti-cancer drugs in various cancers [33,34,35,110]. As CisR cells exhibited EMT phenotypes, we speculate that EMT contributes to the cisplatin resistance mechanism of OC cells.

Twist has been reported to be involved in the development of acquired chemoresistance, leading to a poorer progression in various human cancer [111,112,113,114,115]. Some study suggested that Twist promotes platinum resistance in ovarian cancer via activation of collagen type XI alpha 1 (COL11A1), GAS6, L1CAM, and Akt signaling [35,116]. Recently, acquisition of therapeutic resistance in ovarian cancer correlated with Twist, EMT phenotype and micro-RNA [117,118,119,120]. Various approaches were applied to overcome the clinical challenges of metastasis and chemoresistance in OC including nanoparticle delivery of siRNA against Twist [121]. In agreement with previous studies, the present study revealed that Twist knockdown reduced metastasis properties by suppressing CSC-like characteristics, migratory ability and invasiveness in CisR OC cells [28,113]. Twist-deficient CisR OC cells exhibited EMT phenotypic characteristics by increasing E-cadherin and reducing N-cadherin and vimentin expression [122]. Twist knockdown CisR OC cells exhibited reduced DNA repair capacity and increased ER stress mediated cell death. In addition, our studies suggested that Twist knockdown could promote cell death via ER stress pathway. The ER stress markers GRP78, cleaved ATF-6 and CHOP were markedly increased in Twist-deficient CisR cell. Intriguingly, ER stress inhibition markedly rescued cell growth and reversed Twist knockdown-induced cell death. Finally, we limited our experimental analysis of OC cell lines to two types in vitro, which may not reflect the in vivo patient environment. Therefore, we cannot conclude that the majority of OC cancers will be correlated with Twist and ERstress-mediated cell death. 

## 4. Materials and Methods

### 4.1. Cell Lines

Human serous OC cell line OV-90 and human epithelial OC cell line SKOV-3 were obtained from Korean Biotech Co., Ltd., Seoul, Korea the domestic distributor of American Type Culture Collection (ATCC). The OV-90 was cultured in a 1:1 mixture of MCDB 105 medium (LM016-01) and Medium 199 (#GIB-11150-059, Gibco, Life Technologies, Grand Island, NY, USA), while SKOV-3 was cultured in McCoy’s 5A modified (#GIB-16600082, Gibco). All media are supplemented with 10% fetal bovine serum (FBS, #GIB-16000-044, Gibco) and 1% penicillin-streptomycin (#P4333, Sigma-Aldrich, St. Louis, MO, USA). Cells were grown in 5% CO_2_ saturated humidity, at 37 °C, and sub-cultured by harvesting with trypsin-ethylenediaminetetraacetic acid (EDTA) (#GIB-25300-054, Life Technologies, Grand Island, NY, USA).

### 4.2. Generation of Acquired CisR Ovarian Cancer (OC) Cell Lines

The acquired CisR OC cell lines were generated by following the previously described method with slight modifications [71,123]. 

### 4.3. CisR Subline 1 (OV-90/CisR1 and SKOV-3/CisR1)

The CisR subline 1 (CisR1) was generated by using cisplatin at pulse treatment at 100 μM for 2 h (20 doses) followed by 100 μM for 4 h (5 doses), 8 h (5 doses), 16 h (5 doses), and 24 h (5 doses). The treated cells were then allowed to grow in drug-free medium. No further treatment was administered until the cells were in exponential phase. 

### 4.4. CisR Subline 2 (OV-90/CisR2 and SKOV-3/CisR2)

The CisR subline 2 (CisR2) was generated by stepwise increasing doses of cisplatin (10, 20, 40, 80 and 100 μM), and each dosage of cisplatin was administered 10 times. The cells were incubated in cisplatin-containing medium and another drug treatment was administered when cells were in exponential phase.

The two resistant sub-clones were established over a period of 8 months. All the resistant cells were maintained in a medium containing 2 μM of cisplatin supplemented with 10% FBS, 1% penicillin and streptomycin. Cells were kept at 37 °C in a humidified atmosphere of 5% CO_2_ and 95% air. These cell lines grew in monolayers and were passaged when cultures were 70–80% confluent. No experiments were performed until all the cells had been maintained in drug-free medium for 1 month.

### 4.5. siRNA Transfection

To create a knockdown of Twist, we transfected cells using Twist siRNA (#sc38604, Santa Cruz Biotechnology, Dallas, TX, USA) and control siRNA (#sc37007). The lyophilized siRNA duplex was reconstituted in RNase-free water to create 10 μM stock solutions. Lipofectamine 2000 RNAiMAX (#13778030, Invitrogen, Waltham, MA, USA) was used to transfect the siRNA into cells according to the manufacturer’s instructions. The transfected cells were incubated for 48 h before experiments. 

### 4.6. Determination of 50% Inhibitory Concentration (IC_50_)

Inhibitory concentrations (ICs) were determined using EZ-cytox cell viability kits (#EZ-1000, DLS-1906, DoGenBio Co., Ltd., Seoul, Korea). The cells (1 × 10^4^ cells/well) were plated in 96-well plates and incubated for 24 h (h). The cells were treated with different concentration of cisplatin (0–100 µM) for parental and cisplatin (0–400 µM) for resistant cell lines. After incubation for 24, 48 and 72 h, EZ-cytox solution was added to each well and incubated for 2 h. Absorbance was then recorded on a microplate reader (Synergy H1; BioTek Instruments, Inc., Winooski, VT, USA) at the wavelength of 490 nm. The IC_50_ values were analyzed using GraphPad Prism software (version 5.0, GraphPad Software Inc., San Diego, CA, USA).

### 4.7. Morphological Evaluation and 3-Dimensional (3d) Spheres Generation

For morphological evaluation, the exponentially growing cells were transferred to a 6-well plate and allowed to adhere in 5% CO_2_ at 37 °C. When the confluency of the cells reached 70 to 80%, the plates were washed with phosphate buffer saline (PBS), fixed in methanol, stained with 0.1% crystal violet, dried and photographed under light microscopy. For 3-dimensional (3d) spheres generation, the OV-90/parental, OV-90/CisR1, OV-90/CisR2, SKOV-3/parental, SKOV-3/CisR1 and SKOV-3/CisR2 were cultured in poly 2-hydroxyethyl methacrylate (Poly-HEMA) coated 12-well plates [124]. Each well was coated with 500 µL of poly-HEMA and cells were seeded at 2 × 10^4^ cells/well for 5 days under standard conditions (37 °C, 5% CO_2_). After the incubation period, cells were photographed under light microscopy.

### 4.8. Colony Formation Assay

Colony formation assay was determined using a clonogenic assay [125]. In brief, the parental and CisR cells were cultured in 6-well plates at low density (~1000 cells per well) for 24 h and then treated with 50 µM cisplatin for 24 h followed by 9 days of recovery. The plates were then washed with PBS and stained with 0.1% crystal violet solution. The cells were washed until no stain was visible, air dried, and photographed. The dye was extracted using 1% sodium dodecyl sulfate (SDS) solution by continuous shaking and then quantified using a spectrophotometer at 570 nm.

### 4.9. Cell Migration Assays

Cell migration assay was performed by using a culture medium-treated 6.5 mm transwell chamber with 8.0 μm pore polycarbonate membranes (#3422, Corning Life Sciences, Corning, NY, USA). In brief, the parental and CisR cells were cultured in 6-well plates and were treated with 50 µM cisplatin for 24 h. Cells were then harvested from cell culture plates by serum-free medium and 2 × 10^4^ cells per 300 µL of serum-free medium plated into the transwell insert, while the bottom chamber was filled with 700 μL medium containing 10% FBS. After incubation in a humidified incubator with 5% CO_2_ at 37 °C for the desired period of times (12 and 24 h), non-migratory cells were scraped off from the top of the transwell using a cotton swab. The cells attached to the bottom side of the membrane were fixed by methanol, stained with 0.1% crystal violet, dried, and photographed under a light microscope. The number of migrating cells was measured using a microplate reader at (Synergy H1; BioTek Instruments, Inc., Winooski, VT, USA) absorbance 570 nm. The calculations of percentage of cell migration, migration speed and average migration speed are given in Table 1.

### 4.10. Cell Invasion Assays

Cell invasion assay was performed by using a culture medium-treated Corning BioCoat Matrigel Invasion Chamber with 8.0 μm pore polycarbonate membranes (#354480, Corning Life Sciences, Corning, NY, USA). In brief, the parental and CisR cells were cultured in 6-well plates and were treated with 50 µM cisplatin for 24 h. Cells were then harvested from cell culture plates by serum-free medium and 2 × 10^4^ cells per 300 µL of serum-free McCoy’s 5A medium was plated into the transwell insert, while the bottom chamber was filled with 700 μL medium containing 10% FBS. After incubation in a humidified incubator with 5% CO_2_ at 37 °C for the desired period of time (12 and 24 h), non-invasive cells were scraped off from the top of the transwell with a cotton swab. The cells attached to the bottom side of the membrane were fixed by methanol, stained with 0.1% crystal violet, dried, and photographed under a light microscope. The number of invading cells was measured by the microplate reader (Synergy H1; BioTek Instruments, Inc., Winooski, VT, USA) at absorbance 570 nm. The equations for calculating the percentage of cell invasion and the invasion speed are given in Table 2.

### 4.11. Wound Healing Assay

Cell mobility was assessed using a scratch wound healing assay. In brief, the parental and CisR cells were cultured in 6-well plates for 24 h and then treated with 50 µM cisplatin for another 24 h. Cells were re-suspended and again 2 × 10^5^ cells were seeded into six-well plates and cultured to monolayers, which were then wounded using sterile 1 mL pipette tips. Cells were washed with PBS to remove any debris. Photos were captured at 0, 12 and 24 h after wounding. The gap distance can be quantitatively evaluated using software such as ImageJ (National Institutes of Health, Bethesda, MD, USA). The equations for calculation of percentage of wound closure, wound healing speed and relative wound area are given in Table 3.

### 4.12. Adhesion Assay

The adhesion assay was performed with the CytoSelect cell adhesion kits, which utilizes a Fibronectin-coated 48-well plate (#CBA-050, Cell Biolabs, Inc., San Diego, CA, USA). In brief, the parental and CisR cells were cultured in 6-well plates for 24 h and then treated with 50 µM cisplatin for another 24 h. Then, under sterile conditions the Fibronectin adhesion plate was allowed to warm up at room temperature for 10 min (min) and a cell suspension containing 1 × 10^6^ cells/mL was prepared in serum-free medium. Then, 150 µL of cell suspension was added to the inside of each well (BSA-coated wells act as a negative control) and incubated for 90 min in cell culture incubator (37 °C, 5% CO_2_ atmosphere). After that, the researchers carefully discarded or aspirated media from the wells and gently washed each well 4–5 times with 250 µL of PBS. Then, the researchers added 200 µL of cell stain solution and incubated for 10 min at room temperature. The cell staining solution was discarded or aspirated from the wells, and each well was gently washed 4–5 times with 500 µL of deionized water and allowed to air dry. After taking photographs of each group, 200 µL of extraction solution was added per well, followed by incubating for 10 min on shaking machine. Next, 150 µL was transferred from each extracted sample to a 96-well microtiter plate, and the OD 570 nm was measured in the plate reader (Synergy H1; BioTek Instruments, Inc., Winooski, VT, USA).

### 4.13. Apoptosis Assay

Hoechst 33,342 staining was conducted to distinguish apoptotic cells from normal cells. In brief, cells were seeded in 6-well plates with 2 × 10^5^ cells per well in culture media and were allowed to attach overnight. The cells were treated with cisplatin at doses of 50 µM and incubated at 37 °C for 24 h. After incubation, the seeded cells were washed on the 6-well plate PBS once, then incubated with 5 μg/mL Hoechst 33,342 for 15 min. Finally, cells were washed twice with PBS and observed using inverted fluorescence microscopy (Axioskop 2 plus microscope, Carl Zeiss, Oberkochen, Germany). The apoptotic nuclei were counted from five non-overlapping fields and expressed as a percentage of the total number of nuclei counted.

### 4.14. Western Blotting

Total protein for Western blotting was extracted from cells using Pro-Prep protein extraction solution (#17081, iNtRON Biotechnology, Seongnam, Korea) including protease inhibitor cocktail (#P8340, Sigma-Aldrich, St. Louis, MO, USA), phosphotase inhibitor cocktail 2 (#P5726, Sigma-Aldrich, St. Louis, USA) and phosphotase inhibitor cocktail 3 (#P0044, Sigma-Aldrich, St. Louis, MO, USA), centrifuged at 11,000× *g* for 15 min, and quantified using a bicinchoninic acid (BCA) assay kit (#21071, iNtRON Biotechnology, Seongnam, Korea). The proteins were separated by 8 to 15% using sodium dodecyl sulfate-polyacrylamide (SDS-PAGE)_gel electrophoresis and transferred to 0.2 µM polyvinylidene difluoride (PVDF) membranes (#IB24001, Invitrogen; Thermo Fisher, Carlsbad, CA, USA) using the iBlot 2 dry blotting system (Invitrogen; Life Technology; Carlsbad, CA, USA). The primary and corresponding secondary antibodies were incubated in the iBind Western Device (Thermo Fisher Scientific, Carlsbad, CA, USA) using iBind solution kits (#INV-SLF1020, Thermo Fisher Scientific, Carlsbad, CA, USA). The primary antibodies were: mouse monoclonal anti-XRCC1 (#MA5-12071, Thermo Fisher Scientific, Carlsbad, CA, USA), mouse monoclonal anti-PARP-1 (#SC-8007, Santa Cruz Biotechnology, Dallas, TX, USA), rat monoclonal anti-GRP78 (#SC-13539, Santa Cruz Biotechnology), mouse monoclonal anti-ATF-6α, (#SC-16659, Santa Cruz Biotechnology), mouse monoclonal anti-CHOP (#2895, Cell Signaling Technology. Danvers, MA, USA), mouse monoclonal anti-Bax (#SC-20067, Santa Cruz Biotechnology), mouse monoclonal anti-Bcl-2 (#SC-509, Santa Cruz Biotechnology), rabbit anti-cleaved caspase-9 (ab2324, Abcam, Cambridge, MA, USA), rabbit polyclonal anti-cleaved caspase-3 (#9661, Cell Signaling Technology, Danvers, MA, USA), mouse monoclonal anti-E-cadherin (#SC-8426, Santa Cruz Biotechnology), mouse monoclonal anti-N/R-cadherin (#SC-81417, Santa Cruz Biotechnology), mouse monoclonal anti-Twist (#SC-8426, Santa Cruz Biotechnology), mouse monoclonal anti-vimentin (#SC-8426, Santa Cruz Biotechnology) and mouse monoclonal anti-β -actin (#A5441, Sigma-Aldrich, St. Louis, MO, USA). The proteins were visualized using EZ-Western Lumi Plus solution (#WSE-7120L, ATTO Corporation, Tokyo, Japan) in EZ-Capture ST (ATTO Corporation, Tokyo, Japan), and bands were measured using ImageJ software.

## 5. Conclusions

**Figure 11:**
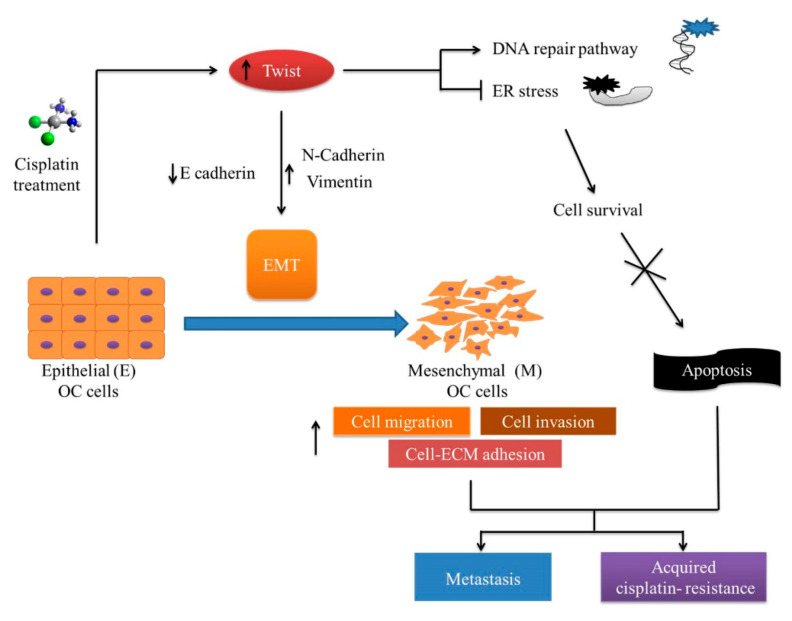
The possible molecular mechanism of acquired cisplatin resistance in ovarian cancer (OC) cells. Prolong or high dose of cisplatin lead to increase expression EMT transcription factor Twist followed by enriched metastasis and acquired chemotherapy resistance. The CisR cells reserved the metastasis properties, including cell proliferation, migration, invasion and cell adhesion. The CisR cells acquire the ability to repair DNA and reduce ER-stress-mediated cell death. The EMT-related phenotypes are strongly expressed in the CisR, which could lead to enhanced metastasis and acquired cisplatin resistance.

The generation and characterization of OC cell lines in this study provide an important scientific resource for studying the molecular mechanism of acquired chemotherapy resistance in ovarian cancer therapy. The individual signature (Figure 11) exhibited by the CisR cell model offers a framework for the development of new molecular targets to treat ovarian cancer disease. In particular, the role of Twist and ER-stress-mediated cell death could allow us to understand the molecular mechanism of complex acquired cisplatin resistance, and develop a new strategy to overcome CisR OC.

## Figures and Tables

**Table 1:** Calculations for measuring cell migration.

Parameter	Calculation
Cell migration (%)	A_CisR_/A_p_ × 100
Cell migration speed (%/h)	M/∆T

A_CisR_, absorbance of cisplatin-resistant cells at 570 nm; A_p_, absorbance of parental cell at 570 nm; M, migration (%); ∆T, duration of migration (h).

**Table 2:** Calculations for measuring cell invasion.

Parameter	Calculation
Cell invasion (%)	A_CisR_/A_p_ × 100
Cell invasion speed (%/h)	I/∆T

A_CisR_, absorbance of cisplatin-resistant cells at 570 nm; A_p_, absorbance of parental cell at 570 nm; I, invasion (%); ∆T, duration of invasion (h).

**Table 3:** Calculations for measuring wound healing.

Parameter	Calculation
Would closure (%)	W_0_ − W_t_/W_0_ × 100
Healing speed (µm^2^/h)	W_0_ − W_t_/∆T
Relative wound area	W_0_/W_t_

W_0_, wound area at 0 h (µm^2^); W_t_, wound area at ∆h (µm^2^), ∆T, duration of wound measured (h).

## Supplementary Materials

The following are available online at https://www.mdpi.com/1422-0067/21/20/7613/s1: Figure S1: The 50% inhibitory concentration (IC_50_) value of cisplatin in parental and CisR OC cells; Figure S2: The migration capability of parental and CisR OC cells assessed by transwell migration assay; Figure S3: The migration capability of parental and CisR OC cells assessed by scratch wound healing assay; Figure S4: The invasion capability of parental and CisR OC cells assessed by transwell migration assay; Figure S5: The adhesion capability of parental and CisR OC cells assessed by fibronectin-adhesion assay; Figure S6: The expression of DNA repair proteins, PARP1 and XRCC1 to relative beta actin in parental, siTwist and siNC cells; Figure S7: Twist knockdown induces the expressions of ER stress proteins, GRP78, cleaved ATF-6 and CHOP relative to beta actin in parental, siTwist and siNC cells; Figure S8: Twist knockdown induces cell death protein expression in CisR OC cells.
