# Supplementary material for: Establishment of Acquired Cisplatin Resistance in Ovarian Cancer Cell Lines Characterized by Enriched Metastatic Properties with Increased Twist Expression
Source: Int J Mol Sci. 2020 Oct 15;21(20):7613. doi: 10.3390/ijms21207613 (PMC7589258; doi:10.3390/ijms21207613)
Supplement: Supplementary file 1 [file ijms-21-07613-s001.pdf]

Supplementary Materials: Establishment of Acquired Cisplatin Resistance in Ovarian Cancer Cell Lines Characterized by Enriched Metastatic Properties with Increased Twist Expression

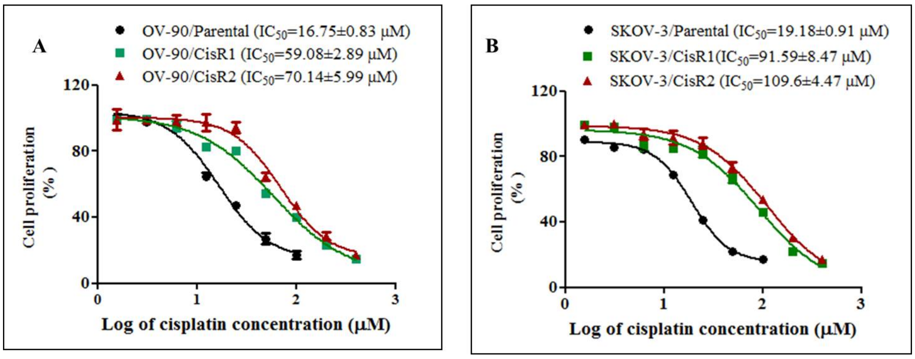

Figure S1. The 50 % inhibitory concentration (IC_{50}) value of cisplatin in parental and CisR OC cells at 72 h. (A) OV-90 and (B) SKOV-3 cell lines. Values were represented as mean±SD.

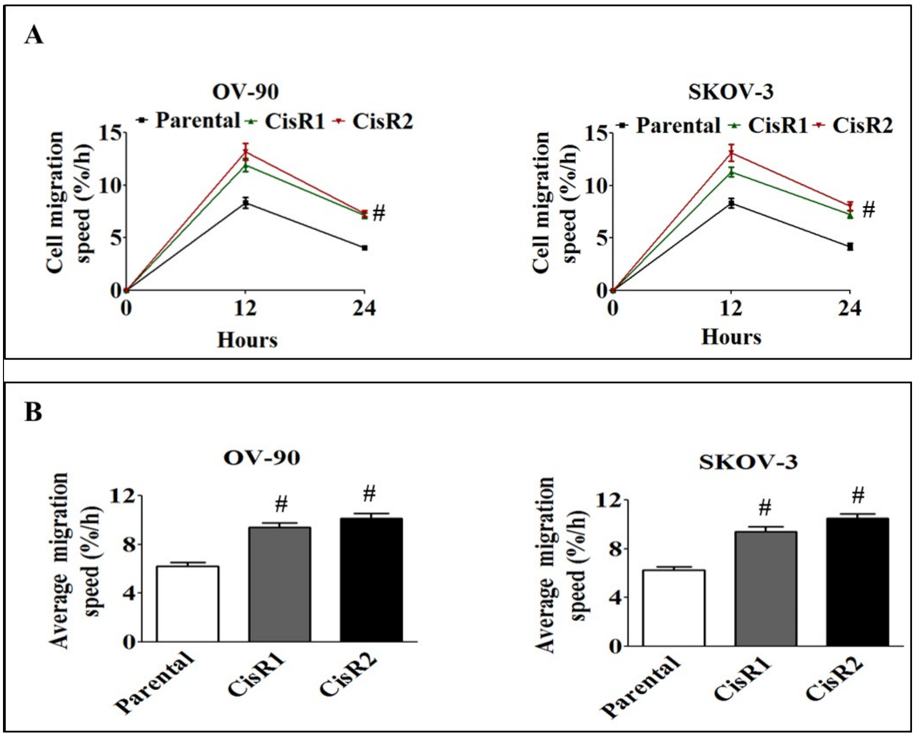

Figure S2. The migration capability of parental and CisR OC cells assessed by transwell migration assay. (A) Cell migration speed and (B) Average cell migration speed measurement in OV-90 and SKOV-3 cell lines. Values were represented as mean±SD. $*p<0.05$, $\#p<0.01$, compared with the parental group.

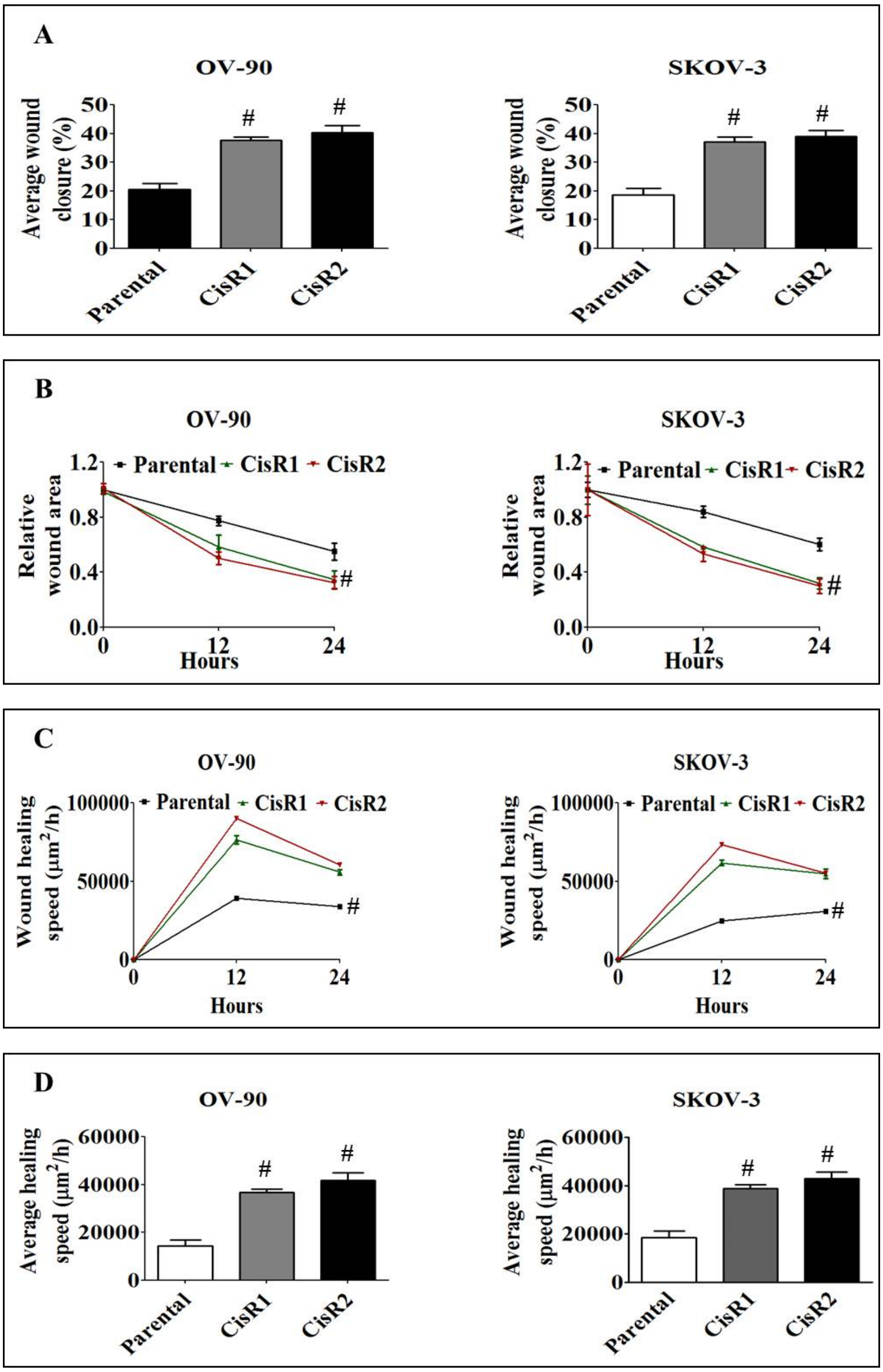

Figure S3. The migration capability of parental and CisR OC cells assessed by scratch wound healing assay. (A) Average wound closure percentage and (B) relative wound area. (C) Wound healing speed (D) Average wound healing speed measurement in OV-90 and SKOV-3 cell lines. Values were represented as mean±SD. *$p<0.05$, #$p<0.01$, compared with the parental group.

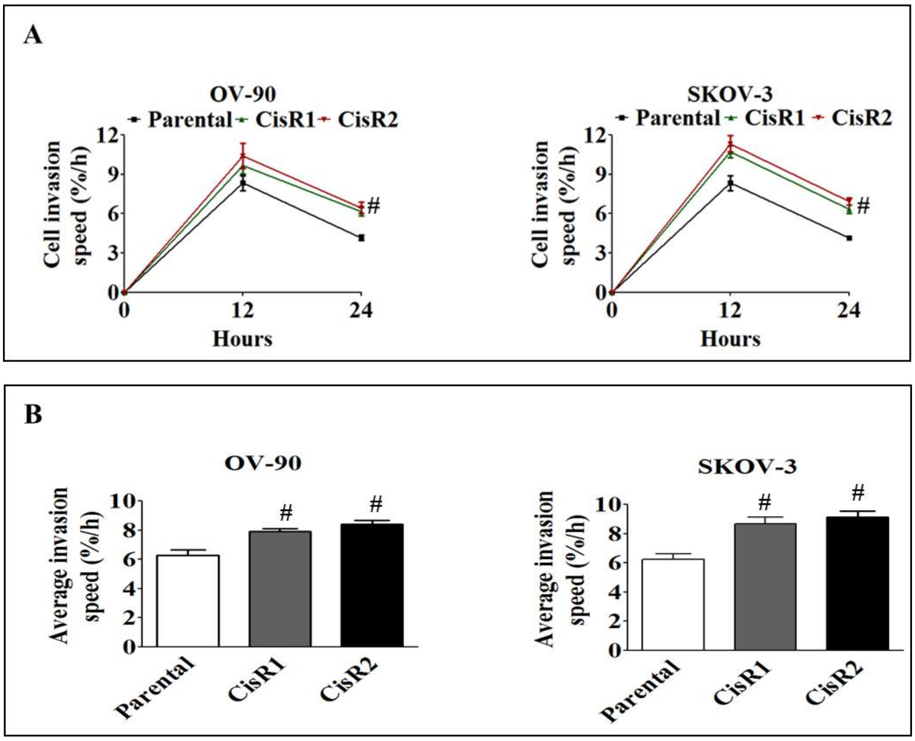

Figure S4. The invasion capability of parental and CisR OC cells assessed by transwell migration assay. (A) Cell invasion speed and (B) Average cell invasion speed in OV-90 and SKOV-3 cell lines. Values were represented as mean±SD. *p<0.05, #p<0.01, compared with the parental group.

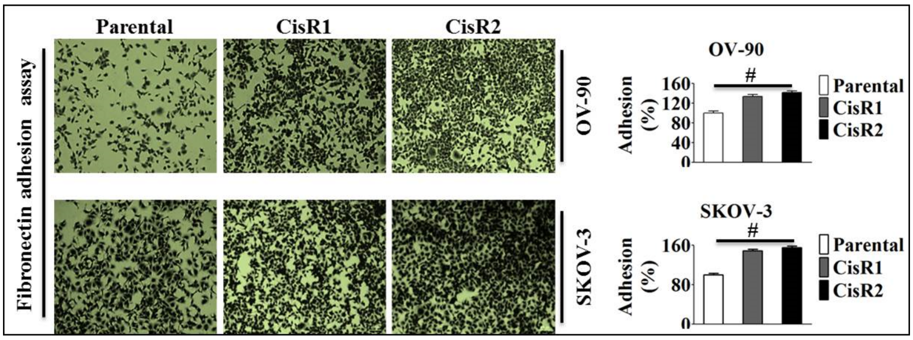

Figure S5. The adhesion capability of parental and CisR OC cells assessed by fibronectin-adhesion assay (Magnification, 10x, scale bar 100 μm). Values were represented as mean±SD. *p<0.05, #p<0.01, compared with the parental group.

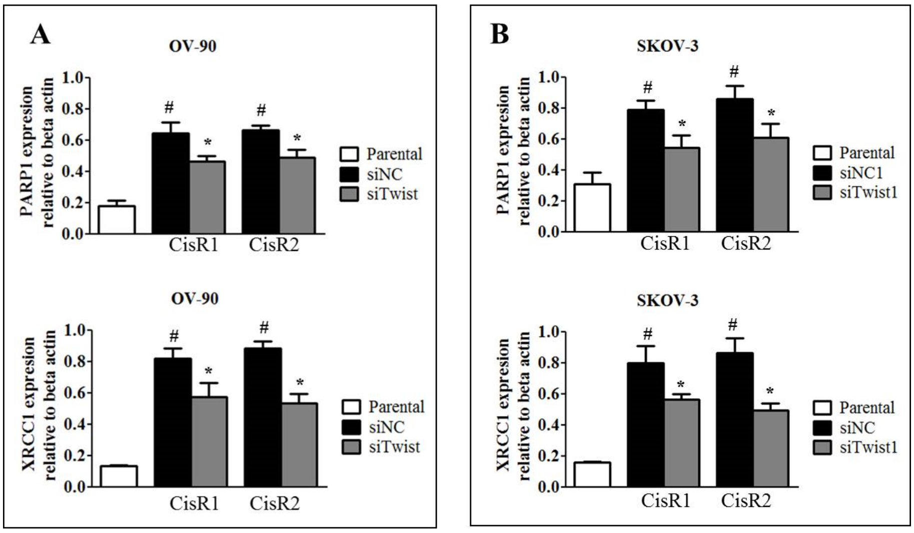

Figure S6. The expression of DNA repair proteins, PARP1 and XRCC1 to relative beta actin in parental, siTwist and siNC cells. (A) The protein expression in OV-90, OV-90/CisR1 and OV-90/CisR2 cells. (B) The protein expression in SKOV-3, SKOV-3/CisR1 and SKOV-3/CisR2 cells. Parental: Non-transfected parental cells; siNC: Cisplatin resistance cells transfected with non-targeting negative control siRNA; siTwist: Cisplatin resistance cells transfected with Twist siRNA. Values were represented as mean±SD. $^{*}p<0.05$, $^{\#}p<0.01$, compared with the parental group.

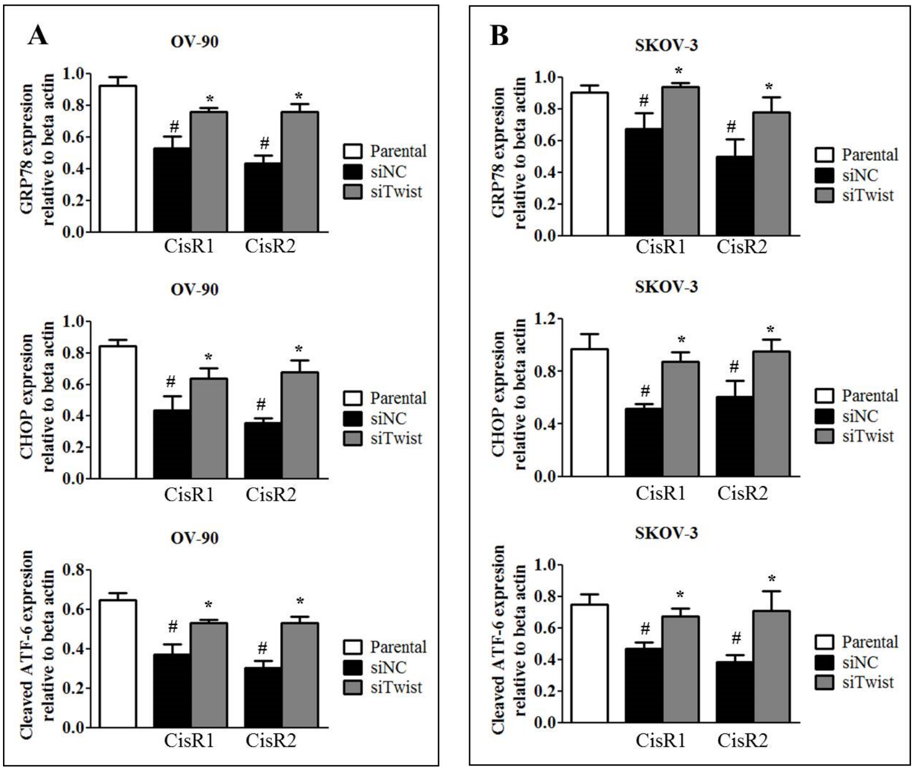

Figure S7. Twist knockdown induces the expressions of ER stress proteins, GRP78,

cleaved ATF-6 and CHOP relative to beta actin in parental, siTwist and siNC cells. (A) The protein expression in OV-90, OV-90/CisR1 and OV-90/CisR2 cells. (B) The protein expression in SKOV-3, SKOV-3/CisR1 and SKOV-3/CisR2 cells. Parental: Non-transfected parental cells; siNC: Cisplatin resistance cells transfected with non-targeting negative control siRNA; siTwist: Cisplatin resistance cells transfected with Twist siRNA. Values were represented as mean±SD. #p<0.05, compared with the parental group and *p<0.05, compared with siNC group.

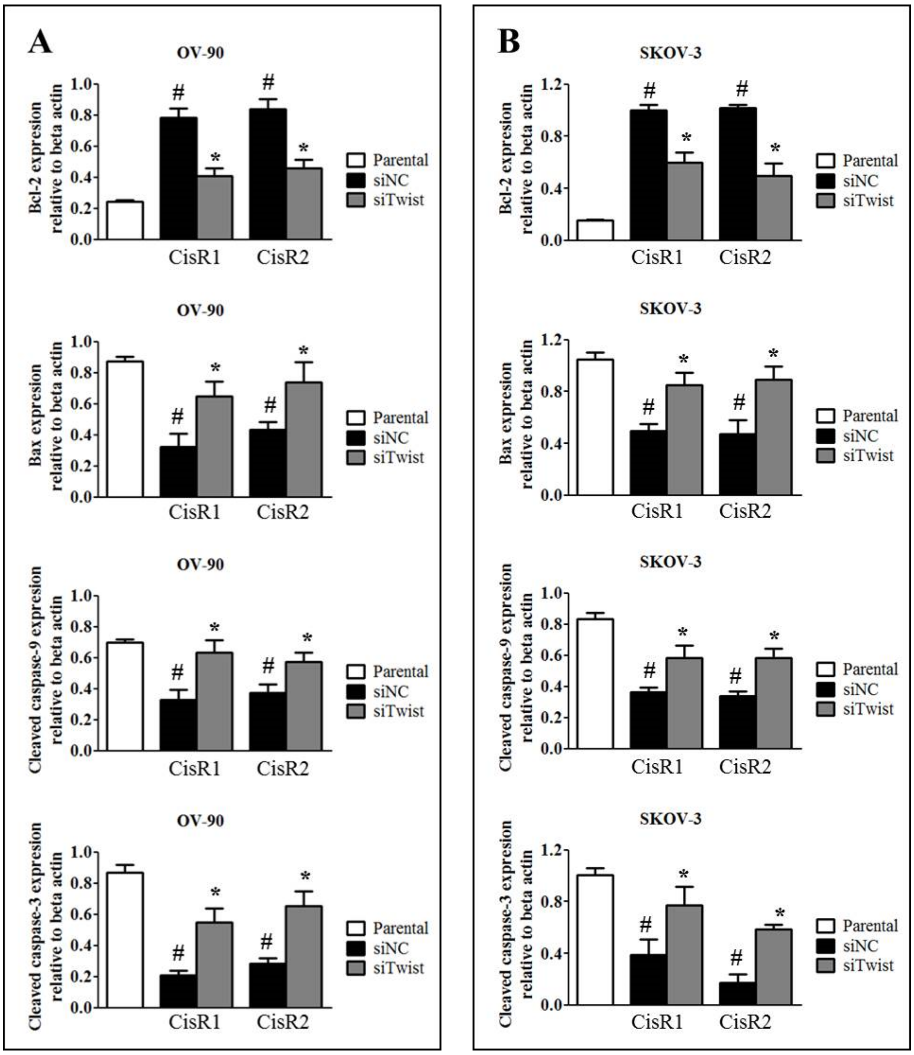

Figure S8. Twist knockdown induces cell death protein expression in CisR OC cells. The expression of apoptotic (Bax, cleaved caspase-9 and cleaved caspase-3) and anti-apoptotic protein proteins (Bcl-2) relative to beta actin in parental, siTwist and siNC cells. (A) The protein expression in OV-90, OV-90/CisR1 and OV-90/CisR2 cells. (B) The protein expression in SKOV-3, SKOV-3/CisR1 and SKOV-3/CisR2 cells. Parental: Non-transfected parental cells; siNC: Cisplatin resistance cells transfected with non-targeting negative control siRNA; siTwist: Cisplatin resistance cells transfected with Twist siRNA. Values were represented as mean±SD. #p<0.05, compared with the parental group and *p<0.05, compared with siNC group.
